# Incentivized self-vaccination for global measles eradication

**DOI:** 10.1016/j.jve.2022.100310

**Published:** 2022-12-15

**Authors:** Benjamin L. Sievers, Robert E. Sievers, Eric L. Sievers

**Affiliations:** a J. Craig Venter Institute, La Jolla, CA, 92037, USA; b Department of Chemistry and Biochemistry, University of Colorado Boulder, Boulder, CO, 80309, USA; c BioAtla Incorporated, San Diego, CA, 92121, USA

## Abstract

Measles–we've become inured to its cruel, insidious impact as it kills over 100,000 children yearly because of suboptimal vaccination coverage. It does not have to be this way. A familiar, safe, exceptionally effective measles vaccine saves lives and permanent, global measles eradication is within reach. But now we need to be clever and courageously explore new strategies to save lives. Firstly, let us enable people to vaccinate themselves, not with a needle and syringe, but with a quick inhaled puff of dry powder vaccine. Secondly, let us provide micro-payments using digital currency to incentivize those who vaccinate themselves. Thirdly, let us leverage learnings from how our social networks guide our behaviors to further encourage self-vaccination. Fourthly, let us inspire friendly regional competition among communities vying for the highest proportion of citizens who show measles neutralizing antibodies in spot saliva samples. With global cooperation and relentless determination, we eradicated smallpox. Next up? Measles.

Since 1963, by employing a safe, highly-protective injected measles vaccine in coordinated global mass vaccination campaigns, we came achingly close to globally eradicating measles in 1995, then again in 2007, and again in 2012.^1^, ^2^, ^3^ But measles did not disappear as smallpox did following a similarly comprehensive global vaccination campaign. Instead, more than 100,000 preventable deaths continue to occur yearly, predominantly among young children living in low and middle-income countries. Missed vaccinations due to the COVID pandemic have further amplified an ongoing crisis.^4^

What happened? Despite diligent yearly vaccination campaigns, global coverage with the first dose of measles-containing vaccine has not budged beyond 86% for the first dose and 60% for the second dose for the past five years.^5^ To vanquish measles, it is estimated that we need to ensure that two doses are delivered and we need to reach 95% seropositivity for sustained intervals.^6^ Notably, the same remote regions most distant from centralized vaccination centers remain woefully unprotected year after year, despite well-intentioned efforts to better target vaccine distribution with catch-up measles vaccination campaigns to those most in need.^6^, ^7^, ^8^, ^9^

The “top-down” centralized approach has protected innumerable lives and is amply celebrated. However, to eradicate measles we now need a new approach. Let us enable people to vaccinate themselves. Let us leverage positive social network confirmation from friends and neighbors to amplify community acceptance of self-vaccination. Let us pay people a little bit of money to vaccinate themselves. Let us collect saliva so we can track measles antibody levels to monitor our collective progress. And let us begin among regions such as in Zimbabwe and Samoa where preventable measles outbreaks have actively killed children.^10^^,^^11^

When most of us visualize vaccination, we imagine waiting in crowded healthcare settings, a needle, a jab, and perhaps some pain. However, many vaccines are now amenable to needle-free self-administration by inhalation, ingestion, or transdermal application.^12^ Well over 150 million doses of a live-attenuated strain of Salmonella typhi (Ty21a) vaccine have been safely consumed orally in the absence of direct supervision by a healthcare provider.^13^^,^^14^ In a randomized, prospective study of 1077 military volunteers, influenza vaccine self-inhaled intranasally showed an acceptable safety and immunogenicity profile and was preferred by volunteers compared to inhaled vaccine administered by a healthcare worker.^15^ In a smaller, single-arm trial, 96% of volunteers who were taught how to inhale the vaccine into their nose believed that they performed the vaccination correctly and preferred self-vaccination to that delivered by a provider.^16^ Similar preferences for self-vaccination were observed by those who self-vaccinated with an investigational intradermal influenza vaccine.^17^^,^^18^

Such an approach could be used with measles. Over the past six decades, subcutaneous injections of the measles vaccine have safely protected the vast majority of humans on the planet from measles infection. Through a centralized inhalation vaccination campaign spearheaded by Albert Sabin in Mexico in the 1980s, nearly 4 million children safely achieved lifetime measles protection through needle-free, aerosolized wet mist vaccination.^19^, ^20^, ^21^, ^22^ Vaccine efficacy was 92%, comparable to the efficacy observed by subcutaneous injection.^23^ More recently, a large, prospective randomized trial demonstrated that aerosolized wet mist live attenuated measles vaccine in children from 9.0 to 11.9 months of age elicited immunogenicity in 85.4%; (95% confidence interval [CI], 82.5 to 88.0) versus 94.6%; (95% CI, 92.7 to 96.1) for those young children who had received the conventional subcutaneous vaccine.^24^ It is possible that comprehensive, real-world protection against measles by aerosolized vaccine was underestimated by the surrogate endpoint of neutralizing antibodies. Importantly, cell-mediated immunity was not evaluated in the randomized trial. Conceivably, infection of the airways from the attenuated inhaled measles vaccine likely induced an integrated protective mucosal response that wasn not characterized with a simple immunogenicity endpoint.

Aerosolized vaccine provided as a booster might be particularly protective for measles among older children and adults. Promisingly, when given as a second dose, inhaled measles vaccine achieved higher and more sustained levels of protection compared with subcutaneously administered vaccine.^25^ Taken together, aerosolized vaccine enabled remarkably robust protection against measles infection through a single inhalation, obviating the need for syringes and needles though still requiring skilled health care providers, reconstitution of the vaccine with water, and an energy source to power the nebulizer.

But a needle-free, temperature-stable, dry powder, live-attenuated inhaled measles vaccine is now available.^26^ A novel spray drying vaccine manufacturing method employing carbon-dioxide-assisted nebulization creates exceptionally fine particles ranging from 3 to 5 μm that maintain the potency of living attenuated live virus vaccine and enable aerosolized delivery to the deep lung through inhalation. In cotton rats, the inhaled measles vaccine was protective, producing potent neutralizing antibodies as shown through plaque reduction neutralization assays.^27^ In preclinical studies, delivering the aerosolized dry powder by deep inhalation, the vaccine induced measles virus-specific humoral and T cell responses in rhesus macaques without adverse effects.^28^^,^^29^ More than one year later, the macaques were fully protected from infection with wild-type measles virus.^30^ Subsequently, the live-attenuated dry powder inhalable measles vaccine was shown to be well-tolerated in a phase I clinical trial in healthy measles seropositive human volunteers.^31^ Based upon these findings, it is now envisioned that dry powder measles vaccine doses in a metered-dose inhaler can be further evaluated for delivery by vaccination inhalation whistles for adults and older children or a whistle face mask attachment for younger children to enable global self-vaccination strategies.^32^

To self-vaccinate, sociological and behavioral challenges highly relevant to the goal of achieving global measles eradication. We believe that broad adoption of self-vaccination by individuals will be positively influenced by the ease of use and by what people's peer networks think of such a novel approach. Social contagions come in two flavors: simple and complex. Unlike the simple transmission of the measles virus from one unvaccinated person to the next, we anticipate an individual's specific willingness to inhale a measles vaccination on their own terms will follow a so-called complex contagion model.^33^^,^^34^ We believe that the willingness to self-vaccinate in sufficiently mature subjects will meaningfully increase through social reinforcement, akin to choosing to wear a mask when in poorly ventilated spaces. To succeed, we will need to integrate evidence-based social networking approaches by specifically targeting and educating influential community members with the broader goal of achieving sustainable community-wide self-vaccination practices.

Attaining a critical adoption threshold of at least 25% has also been shown to be associated with subsequent acceleration of target behaviors by the larger group and is likely to play a key role in disseminating self-vaccination throughout a community.^35^ Plus, a financial incentive has the potential to further cultivate self-vaccination behaviors in the context of encouragement from family and peers. In a prospective trial performed in Tanzania, the provision of modest financial payments improved compliance with viral suppression medications for adults early in their HIV treatment. Providing either the equivalent of approximately $4.50 or $10.00 in local currency to research participants was associated with treatment compliance improving from a baseline rate of 73% to either 83% or 86%, respectively.^36^

Those who self-vaccinate could be incentivized through wireless provision of a digital vaccine coin employing existing decentralized finance systems among countries where this approach is broadly familiar. This secure, blockchain approach is associated with an added benefit of enabling individual vaccine safety monitoring akin to the Vaccine Adverse Event Reporting System (VAERS) used in the United States. Conceivably, depending upon regional vaccination target goals and support from governmental and/or commercial partners, a newly-vaccinated individual's fungible vaccine coin could be traded for valued services like upgraded mobile phone functionality or streaming video.

Additionally, cultivating friendly, convivial competition between local regions to achieve the highest protective measles seropositivity rates holds the additional potential to vanquish outbreaks. In one example from Rotterdam, a prospective program cultivated friendly competition between nine hospitals and one rehabilitation center resulting in an increased incidence of handwashing behaviors by healthcare providers.^37^ We believe the goal of decreasing preventable measles-associated deaths in young children has the potential to inspire helpful competition between regions seeking to maximize measles seropositivity rates that can be objectively measured regularly.

Global measles eradication is attainable if we diversify our vaccination strategies. Let us integrate incentivized self-vaccination into existing mass immunization campaigns. Imagine a brightly-colored vaccine whistle in simple packaging festooned with pictorial instructions. Imagine simply taking one deep inhalation. As you hear the whistle's tone, you know the vaccine has been delivered and that measles will not be able to use your body to infect others. Your vaccination experience is memorable in its simplicity. You did not wait in a healthcare facility and there were no needles. You encourage your family and friends to self-vaccinate. And the whistle is yours to keep.

## Declaration of competing interest

The authors declare that they have no known competing financial interests or personal relationships that could have appeared to influence the work reported in this paper.

## Data Availability

No data was used for the research described in the article.
